# Fluoroquinolone Resistance and *Clostridium difficile*, Germany

**DOI:** 10.3201/eid1604.090859

**Published:** 2010-04

**Authors:** Nils Henning Zaiß, Wolfgang Witte, Ulrich Nübel

**Affiliations:** Robert Koch Institute, Wernigerode, Germany

**Keywords:** Clostridium difficile, bacteria, Germany, fluoroquinolone resistance, enteric infections, dispatch

## Abstract

We characterized 670 *Clostridium difficile* isolates collected from patients in 84 hospitals in Germany in 2008. PCR ribotyping showed high prevalence of ribotype 001 and restricted dissemination of ribotype 027 strains. Fluoroquinolone resistance and associated gyrase mutations were frequent in various ribotypes, but no resistance to metronidazole or vancomycin was noted.

During the past decade, incidence rates of *Clostridium difficile* infections (CDI) have increased noticeably worldwide (*1*). In the United States and Canada, this increase has been associated with the emergence of a possibly more virulent strain designated North American pulsotype 1 (NAP1), or PCR ribotype 027. Strains with identical typing patterns have also been reported from several countries in Europe (*1*). In Germany, the first clusters of infections with *C. difficile* ribotype 027 were identified in the southwest region in 2007 (*2*,*3*). Although incidence rates increased in Germany after 2000 (*4*), an association with particular strains remains unclear, and no nationwide surveillance data on *C. difficile* genotype prevalence exist.

We report the distribution and associated antimicrobial drug susceptibility of prevalent *C. difficile* strains in Germany. Isolates were characterized by PCR ribotyping (*5*) by using the ribotype nomenclature of the Cardiff Anaerobe Reference Laboratory (Cardiff, Wales, UK). Antimicrobial drug susceptibility to therapeutic drugs (metronidazole and vancomycin) and to fluoroquinolones (moxifloxacin and levofloxacin) was determined by using the Etest method (breakpoints according to European Committee on Antimicrobial Susceptibility Testing guidelines).

## The Study

**Figure 1:**
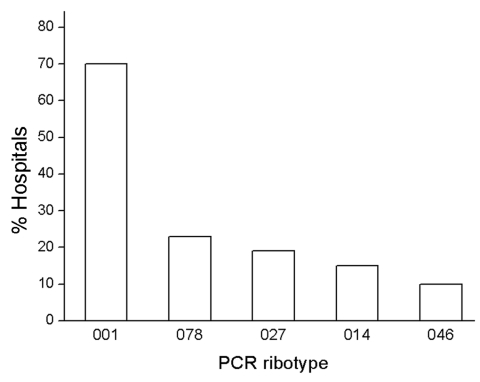
Prevalent PCR ribotypes of *Clostridium difficile* in hospitals in Germany, 2008. Eighty-four hospitals sent isolates from patients with severe *C. difficile* infections to the Robert Koch Institute. Ribotype 001, responsible for severe infections in 59 hospitals (70%), was the most prevalent ribotype, followed by ribotype 078 (19 hospitals, 23%), ribotype 027 (16 hospitals, 19%), ribotype 014 (13 hospitals, 15%), and ribotype 046 (8 hospitals, 10%).

A surveillance study, performed from January through December 2008, reported 5,640 CDI cases in a sample of 1.6 million patients in hospitals in Germany (www.nrz-hygiene.de). Six percent of cases were defined as severe CDI based on the following criteria: readmission to a healthcare facility due to recurrent CDI, admission to an intensive care unit, surgical intervention (colectomy), or death within 30 days after diagnosis. By projecting these case rates to all of Germany (17 million patients 2008; www.destatis.de), we estimated 58,000 CDI cases (including 3,500 severe cases) in 2008. The 670 isolates investigated in this study, which caused severe infections in 84 hospitals throughout Germany in 2008, represent ≈20% of those severe CDI cases. Among these isolates, 57 ribotypes were identified, and 312 isolates were characterized as PCR ribotype 001 (47%), followed by 53 (8%) isolates each of ribotypes 078 and 027. Figure 1 shows the distribution of the 5 most common ribotypes in proportion to the number of submitting hospitals.

**Figure 2:**
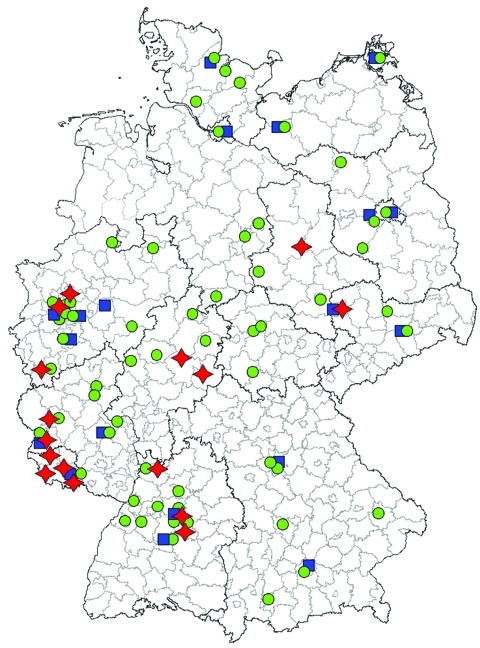
Approximate geographic dissemination of PCR ribotype 001, 078, and 027 of *Clostridium difficile* in hospitals in Germany in 2008. Green dots indicate hospitals with *C. difficile* ribotype 001 infections, blue squares hospitals with ribotype 078 infections, and red stars hospitals with ribotype 027 infections.

Ribotype 001 was by far the most prevalent ribotype found, causing severe CDI in 70% of all collaborating hospitals (Figure 1). A high prevalence of ribotype 001 was also reported in a recent study investigating 2 hospitals in southern Germany (*6*). As depicted in Figure 2, strains of ribotype 001 are endemic to hospitals all over Germany. In contrast, the dissemination of ribotype 027 strains, which could be identified in 16 hospitals (19%), is restricted mostly to the southwest region of Germany; only 2 sporadic cases were observed in the eastern region (Figure 2). The second most prevalent ribotype was 078 (23%). An increasing incidence of infections with 078 was observed in several European countries, and a zoonotic source is widely debated (*7*). Isolates of ribotype 014 and 046 were identified in 15% and 10% of all participating hospitals respectively and are widespread in neighboring countries (*7*).

Antimicrobial drug susceptibility testing showed that all tested isolates were susceptible to metronidazole and vancomycin. The mean MICs for metronidazole and vancomycin were calculated for each of the 7 most common ribotypes (Table 1). In contrast to a previous study from the United Kingdom, there were no significant differences in the mean MICs of metronidazole of common ribotypes 001, 078, and 027 compared with ribotypes 014, 046, 012, and 015 (p = 0.12, Student unpaired *t* test) (*8*).

**Table 1:** Mean MICs of *Clostridium difficile* PCR ribotypes for metronidazole and vancomycin

PCR ribotype (no.)	Metronidazole, mean MIC, mg/L	Vancomycin, mean MIC, mg/L
001 (303)	0.056	1.54
078 (46)	0.071	1.6
027 (51)	0.06	1.42
014 (21)	0.05	1.58
046 (14)	0.041	1.55
012 (14)	0.054	1.96
015 (6)	0.071	1.92

Exposure to fluoroquinolones was described as an independent risk factor for CDI (*9*). Type 001 and 027 isolates from Germany showed widespread resistance to moxifloxacin and levofloxacin (Table 2). In several studies, acquisition of fluoroquinolone resistance in *C. difficile* has been associated with mutations in the active site of DNA gyrase (*10*–*12*). Sequence analysis of subunits *gyrA* and *gyrB* showed that most drug-resistant isolates (including ribotypes 001, 078, 027, 014, and 046) shared the same single transition mutation (ACT to ATT) in *gyrA*, resulting in the amino acid substitution Thr-82→Ile (Table 2). One isolate of type 001 possessed a Thr-82→Ile and Asp-71→Glu (GAC to GAA) mutation and 1 isolate of type 078, resistant to levofloxacin (MIC >32 mg/L) but susceptible to moxifloxacin (MIC = 2 mg/L), showed a Thr-82→Ala (ACT to GCT) change. In addition, high-level resistance (MIC >32 mg/L) to both fluoroquinolones was found in 2 isolates of type 014 and 078 associated with mutations in *gyrB*, although no mutations in gyrA were observed.

**Table 2:** Resistance to moxifloxacin and levofloxacin among the 5 PCR ribotypes of *Clostridium difficile* most common in Germany, 2008

PCR ribotype (no.)	No. (%) isolates, resistant to moxifloxacin (MIC >4 mg/L)	No. (%) isolates, resistant to levofloxacin (MIC >4 mg/L)	Amino acid substitution	
			GyrA	GyrB
001 (303)	301 (99)	301 (99)	Thr-82-Ile, Asp-71-Glu	
078 (46)	29 (63)	30 (65)	Thr-82-Ile, Thr-82-Ala	Asp-426-Asn
027 (51)	51 (100)	51 (100)	Thr-82-Ile	
014 (21)	2 (9)	2 (9)	Thr-82-Ile	Glu-466-Lys
046 (14)	14 (100)	14 (100)	Thr-82-Ile	

The *gyrA* mutations Thr-82→Ala and Asp-71→Glu have not been reported. However, several amino acid substitutions at both positions were noted before, indicating that Thr-82 and Asp-71 in *gyrA* play a major role in conferring fluoroquinolone resistance in *C. difficile (10–12)*. The Thr-82→Ile substitution especially has been associated with fluoroquinolone resistance in C. *difficile* by several groups (*10*–*12*). For instance, Spigaglia et al. described this substitution in 77 fluoroquinolone-resistant isolates affiliated with 19 ribotypes collected in 12 countries in Europe (*10*). Thr-82 in *C. difficile* corresponds to Ser-83 in the quinoline resistance–determining region of *Escherichia coli* (*11*), and mutations at homologous positions have been associated with fluoroquinolones resistance in several bacteria (*13*).

Fluoroquinolone resistance has been suggested to provide a selective advantage for the spread of epidemic *C. difficile* strains (*8*,*9*,*14*). However, resistance arose convergently in *C. difficile* ribotypes as a consequence of selective pressure resulting from widespread fluoroquinolone use. Because identical mutations were found even in many uncommon strains, gyrase mutations and associated fluoroquinolone resistance alone cannot explain the high prevalence of ribotypes 001 and 027.

## Conclusions

We describe the dissemination and antimicrobial drug susceptibility of ribotypes causing severe CDI in Germany in 2008. We found that ribotype 001 was the most prevalent and widespread ribotype, found in 70% of submitting hospitals. Although dissemination of ribotype 027 was more restricted, recent studies in England and Canada demonstrated that ribotype 027 had the ability to replace type 001 within a few years. In England, ribotype 001 had accounted for ≈55% of all CDI cases in the late 1990s but was reduced to 8% in 2008; during the same period, the proportion of ribotype 027 had increased to 41% (*8*). Similarly, studies in Quebec showed a radical change from ribotype 001 strains accounting for 84% of isolates in 2000–2001 to 80% type 027 isolates in 2003–2004 (*14*). This change was associated with increasing rates of CDI illness and death. It therefore seems advisable to increase infection control measures to curb the spread of ribotype 027 in Germany.

Present data suggest that the dissemination of 027 strains in Germany is restricted to the southwest (Figure 2). Therefore, ribotype 027 cannot be the causative agent of increasing nationwide CDI incidence rates. Thus, increased infection control efforts should not be restricted to the exposure of ribotype 027 because severe CDI courses are caused by all toxigenic *C. difficile* strains.

To investigate if other traits (e.g., sporulation) or just random, stochastic events may determine the success of particular strains, a more detailed understanding of *C. difficile* population structure is necessary. Therefore, international investigations based on portable genotyping procedures (*15*) including isolates from both, CDI and asymptomatic carriage, would considerably improve current knowledge.
